# The Study of rs693 and rs515135 in *APOB* in People with Familial Hypercholestrolemia

**DOI:** 10.22074/cellj.2019.5692

**Published:** 2018-11-18

**Authors:** Fatemeh Karami, Iman Salahshourifar, Massoud Houshmand

**Affiliations:** 1 Department of Biology, Science Faculty, Science and Research Branch, Islamic Azad University, Tehran, Iran; 2 Department of Medical Genetics, National Institute for Genetic Engineering and Biotechnology, Tehran, Iran

**Keywords:** *APOB*, Familial Hypercholestrerolemia, Single Nucleotide Polymorphism

## Abstract

**Objective:**

APOB-related familial hypercholesterolemia (FH) is the most common hereditary hyperchlosterolemia with an autosomal dominant pattern. A number of *APOB* variants are the most important risk factors for hyperchlosterolemia. APOB is a large glycoprotein that plays an important role in the metabolism of lipoproteins in the human body. Small changes in the structure and function of *APOB* can cause major problems in lipid metabolism. Two forms of *APOB* are produced by an editing process of gene replication. *APOB48* is required for the production of chylomicrons in the small intestine and APOB100 is essential in liver for the production of very low density lipoprotein (VLDL) and is also a ligand for LDL receptor (LDLR) that mediates LDL endocytosis.

**Materials and Methods:**

In this case-control study, rs693 (in exon 26 of *APOB*) and rs515135 (5 'end of *APOB*) single nucleotide polymorphisms (SNPs) were analyzed in 120 cases of familial hypercholesterolemia and 120 controls. Both SNPs were genotyped by polymerase chain reaction-restriction fragment length polymorphism (PCR-RFLP) where PCR products were digested with specific restriction enzymes recognising each single nucleotide polymorphism.

**Results:**

This study was analyzed by odds-ratio (OR) and its 95% confidence interval (CI) to examine the association of the two SNPs with familial hypercholostermia susceptibility. Statistical analysis showed that both SNPs were in Hardy- Weinberg equilibrium.

**Conclusion:**

We found no significant relationship between rs515135 and familiar hypercholesterolemia. However, there was a significant association between the C allele of rs693 and high familial cholesterol levels. Furthermore, it seems the dominant model of T allele occurrence has a protective role in emergence of disease.

## Introduction

Familial hypercholesterolemia (FH) is a monogenic inherited disorder. The FH heterozygote type has a two- to three-fold increase in low-density lipoprotein (LDL) cholesterol in serum and has a prevalence of 0.2% (at least 1 in 500) in most countries (1-3). The frequency of homozygous FH is 1 in a million and has a six- to eight-fold increase in plasma LDL-cholesterol (LDL-c) with signs appearing in childhood (1, 4). To reduce the risk of atherosclerosis and premature cardiovascular complications, clinical management focuses on early diagnosis of FH (5). The available evidence demonstrates that FH results from a combination of genetic variants and environmental (diet risk factors and tobacco smoking) factors in different populations (3, 6, 7). Genetic predisposition is assumed to be the cumulative result of mutations and/or polymorphisms of genes that may even have a small-effect, leading to a slight increase in LDL-C (8). Detection of heterozygote and homozygote FH in affected family members is an important step for success rate in accurate diagnosis and subsequent family screening (2). 

Given that FH is one of the sole risk factors of coronary heart disease (CHD), identifying FH causing variants and classifying patients into possible FH is important (9). The majority of cases with FH could be explained by genetic mutations in *LDLR, APOB, PCSK9* and *LDLRAP1* (10). *APOB* is the well-known gene that encodes the protein involved in LDL. The defect in apo B-100 receptor binding domain (Arg3500.Gln) is the most prevalent cause of ligand-defective LDL and cause of FH. Mutations or polymorphisms in APOB have been described as causal risk factors of FH (2, 3, 8, 11).

The *APOB* gene is approximately 43kb in size, and consists of 29 exon and is located on the short arm of chromosome 2 (2 p24.1) (12). Susceptible single nucleotide polymorphisms (SNPs), the most common type of genetic variation, are known to be markers of different chromosomal loci in heritable disease (13). Genome- wide association study (GWAS) focuses on cognizance of SNPs as biomarkers of a disease which have been used in numerous biomedical studies (14, 15). As mentioned above, genotyping SNPs helps in the early detection of some patients with genetic susceptibility (6, 13).

The results of the SNP studies must be expounded circumspectly, as these results may be applied exclusively or multiple SNPs can work cooperatively and create a functional difference. Interplay among multiple SNPs may commonly affect the risk of a disease. Evaluation of SNPs may be problematic with respect to SNPSNP interactions, because taking the individual SNPs without considering SNP-SNP interactions hinders the discovery of weak achievements (16-19). The frequency of the clinical phenotype of FH has been estimated at almost 0.002 in the general population, but in some isolated populations, like French Canadians, Finns, Afrikaners, Druze and Lebanese, occurrence of FH can be in a higher-than-normal frequency because of founder effects and de novo mutations in a population (20, 21). Data on allele and genotype frequencies for APOB have been reported For European (22, 23) and Asian (24-26) populations. For example, allele frequencies of *APOB* and the relationship of its genotypes with plasma lipid and lipoprotein levels in the Mongolian Buryat population resembled the Indians but their frequency distribution differed significantly from the Chinese, Malaysians, and Caucasians (25).

At present, the molecular basis of FH has been shown in detail in many populations, but there is still very limited molecular data relating to FH in Iran (20). To investigate associations between the *ApoB* genotype and levels of LDL-C, two SNPs of *APOB*, referred to in more than one study (3, 18, 19), were examined to determine the role of these SNPs in developing FH in Iran. There has been no independent study on the genetic association of rs693 and rs515135 with FH in the Iranian population. Here, we examined the association of these two selected SNPs with increased serum cholesterol and FH. 

## Materials and Methods

One hundred and twenty patients with FH, recruited from Karaj hospital, and 120 healthy persons, as the control group, were included in this study. The clinical characteristics of patients including age, gender, familial heart and brain disease, and familial high cholesterol were collected. Mean age of patients was 48.65 ± 14.02 years. All the participants were informed about the study and signed a written consent form. This study was approved by the Ethical Committee in Karaj Hospital. 

### Blood sample and DNA isolation

Blood samples were collected in tubes containing EDTA (Golden Vac., China). Genomic DNA was extracted with a DNA extraction kit (MBST, Iran) according to the manufacturer’s instructions. The extracted DNA samples were stored in a freezer at -20°C until further use. 

### Genotyping of *APOB* rs693, rs515135 polymorphism

The case and control samples were genotyped for rs515135 and rs693 SNP using polymerase chain reaction- restriction fragment length polymorphism (PCR-RFLP). Oligonucleotide primers for rs693 C>T were:

F: 5´AGA GGA AAC CAA GGC CAC AGT TGC3´
R: 5´TAC ATT CGG TCT CGT GTA TCT TCT3´

and the oligonucleotide primers for rs515135 A>G were:

F: 5´CCT AGT TAA TCC TCA GAA TGA CAC TG3´
R: 5´ ATT GGG GTG GCA ATA GGC GCA AAT TG3´.

PCR amplification was carried out in a total volume of 25 µl consisting of 12.5 µl Master Mix (Tris-HCl, pH=8.5, 1.5 mM MgCl_2_, 0.2% Tween-20, 0.4 mM dNTP, 2 U/µl Amplicon Taq DNA polymerase, stabilizer and inert red (Amplicon Co., Denmark), 0.5 µM of each primer and 100 ng DNA template and ddH_2_O. PCR cycles were an initial denaturation step at 95°C for 4 minutes followed by 35 cycles of denaturation at 95°C for 30 seconds, annealing temperature of 58°C for 30 seconds, extension at 72°C for 1 minute, and a final extension step at 72°C for 10 minutes. 

PCR products were digested with 0.5 µL (10 U) of BglII (Fermentase, Canada) for rs515135 A>G and XbaI (Fermentase, Canada) for rs693C>T at 37°C for 16 hours. The digested fragments were separated on a 2% agarose gel (containing 0.5 µg/ml DNA Staining) and observed under UV light. 

The BglII recognition site is represented by the presence of A allele which produces two fragments of108bp and 261 bp, while the presence of G allele is represented by the remaining uncut fragment of 369 bp. The XbaI recognition site is represented by the presence of T allele which produces two fragments of 26 bp and 110 bp, while the presence of C allele is represented by the remaining uncut fragment of 136 bp. The fragments were separated by 2% agarose gel electrophoresis and then visualized under UV light.

### Sequencing analysis 

**Fig.1:**
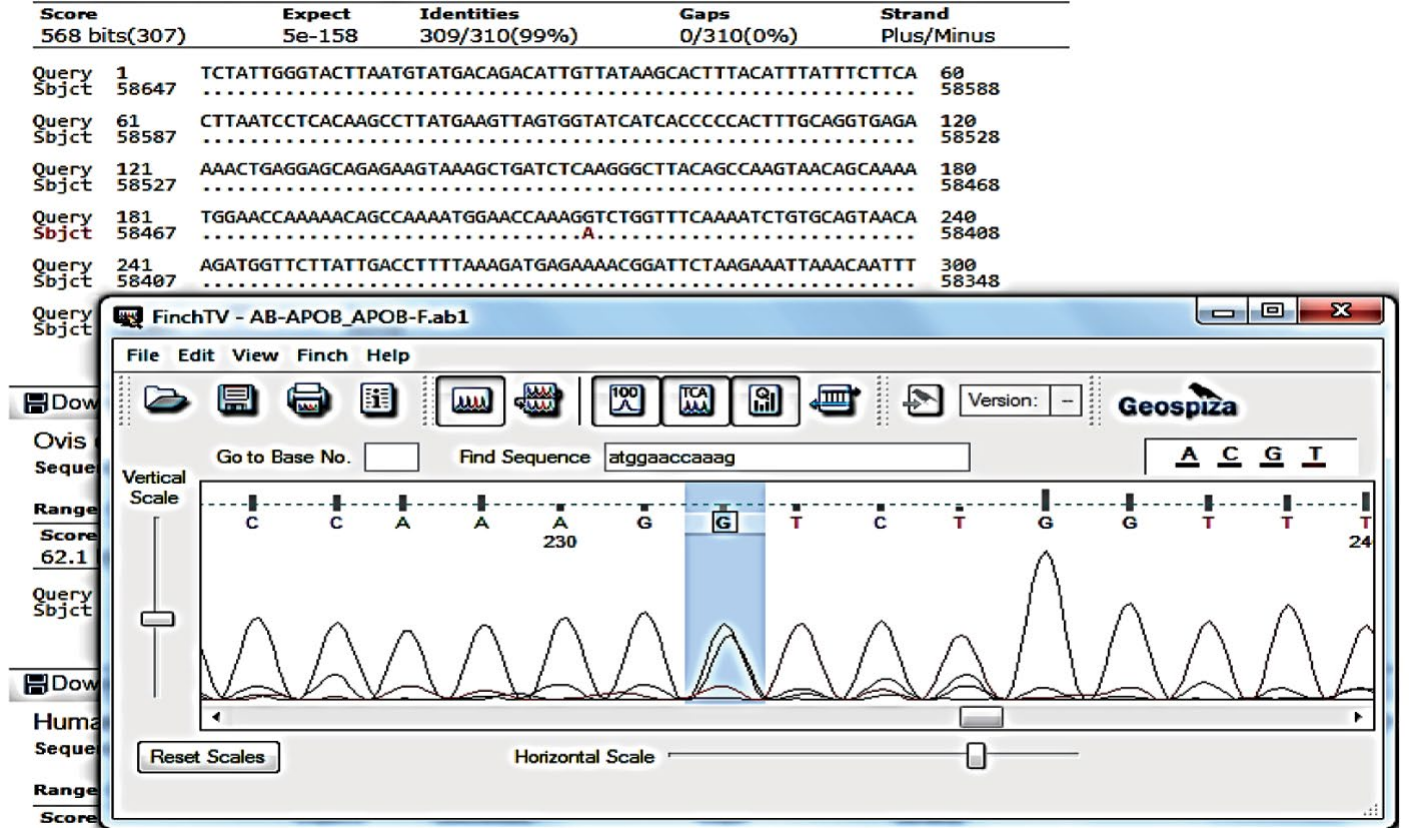
GA genotype of the APOB rs515135 single nucleotide polymorphisms (SNP).

The PCR products were examined for specificity using 2% agarose gel electrophoresis. Double-stranded DNA automated sequencing was performed by using an ABI capillary sequencing machine (Applied Biosystems, gene Fanavaran Company, Iran). All fragments were sequenced with the forward primers. Sequence variants were analyzed using FinchTV (http://www.geospiza. com/finchtv/) (Fig .1).

### Statistical analysis

Statistical analysis was conducted using Graphpad (https://www.graphpad.com/) and Medcalc to perform the chi-square and 95% confidence interval (CI) tests based on APOB rs693 and rs515135 frequencies in FH cases in Iran. A P<0.01 was considered as statistically significant.

## Results

The clinical characteristics of the case and control groups were first compared (Table 1). 

**Table 1:** Demographic characteristics of the FH case group and the healthy control group


Variable		Case	Control	Total	P value
		n=120	n=120	n=240	

Age (Y, mean ± SD)		48.65 ± 14.02	41.35 ± 11.15	-	
Age of disease onset (Y, mean ± SD)		40.46 ± 9.61	NA	-	
Age (Y), n (%)					
	<45	53 (44.16)	79 (65.83)	132 (55)	0.0001
	45-60	41 (34.17)	35 (29.17)	76 (31.67)	
	>60	26 (21.67)	6 (5)	32 (13.33)	
Gender					
	Female	60 (50)	35 (29.17)	95 (39.58)	0.0015
	Male	60 (50)	85 (70.83)	145 (60.42)	
Cholesterol (mg/dl)					
	<200	27 (22.5)	120 (100)	147 (61.25)	
	200-220	66 (55)	0 (0)	66 (27.5)	>0.0001
	>220	27 (22.5)	0 (0)	27 (11.25)	
Familiar heart and brain disease					
	Yes	56 (46.67)	18 (15)	74 (30.83)	>0.0001
	No	64 (53.33)	102 (85)	166 (69.17)	
Familiar high cholesterol					
	Yes	12 (100)	-	-	
	No		NA	-	


NA; Not available and FH; Familial hypercholesterolemia.

To confirm the results of RFLP, 10 samples were sent for sequencing from both SNPs. According to Table 2, in 120 patients group, 84 patients had CC genotype and 36 patients had TT or CT genotypes. In 120 control groups, 103 patients with CC genotype and 17 with TT and CT genotypes were observed. There was a significant correlation between the CC genotype in the patient group and high familial cholesterol (P=0.0037). Table 3 shows the frequency of GG, AA and GA genotypes in patients with high familial cholesterol and the control group. 

### Statistical analysis of rs515135 and rs693 in APOB in the Iranian population

Results of logistic regression analysis showed no significant correlation between genotype and familial high cholesterol disease (Table 4). Regarding the results of logisticregression modeling, there was no significant relationship between genotype and high familial cholesterol patients (P=0.67). Regarding allelic frequencies of rs693 in both groups, the C allele is the prevalent allele in both groups. There was a significant correlation between the C allele and familial high cholesterol. 

**Table 2:** Frequency of the genotype of rs515135 and rs693 in the control group and case group


Genotype		Case n(%)	Control n(%)	Total	P value	OR	95% CI	df

rs515135								
	AA	3 (2.5)	2 (1.67)	5	-	Ref (1)	-	2
	GG	82 (68.33)	85 (70.83)	167	0.6336	1.5549	0.2533-9.5464	
	GA	35 (29.17)	33 (27.5)	73	0.7136	1.4141	0.2221-9.0068	
	Total	120	120	240	-	-	-	
rs693								2
	TT	4 (3.33)	1 (0.83)	5	-	Ref (1)	-	
	CC	84 (70)	103 (85.84)	100	0.1585	4.9048	0.5377-44.74	
	CT	32 (26.67)	16 (13.33)	125	0.5499	2.0000	0.2062-19.3983	
	Total	120	120	240	-	-		


OR; Odd ratio, CI; Confidence interval, and df; Degrees of freedom.

**Table 3:** Frequency of genotype GG+GA and AA in rs515135


Genotype	Patients	Controls	Total	P value	95% CI	df

GG+GA	117 (97.5)	118 (98.33)	135	0.6535	0.2482-9.2201	1
AA	3 (2.5)	2 (1.67)	5			
Total	120	120	240			


CI; Confidence interval and df; Degrees of freedom.

**Table 4:** Frequency of genotype TT+CT and CC rs693


Genotype	Patients	Controls	Total	P value	95% CI	df

CT+TT	36 (30)	17 (141.17)	53			1
CC	84 (70)	103 (85.83)	187	0.0037	1.362-4.949	
Total	120	120	240			


CI; Confidence interval and df; Degrees of freedom.

**Table 5:** Frequency of genotype GA+AA and AA rs515135


Genotype	Case	Control	Total	P value	OR	95% CI	df

GA+AA	38 (31.67)	35 (29.17)	73		Ref (1)		1
GG	82 (68.33)	85 (70.83)	167	0.6739	1.125	0.6491-1.9514	
Total	120	120	240				


OR; Odd ratio, CI; Confidence interval, and df; Degrees of freedom.

Comparison of variables such as age, sex, cholesterol and history of cardiovascular disease in both control and patient groups showed significant differences. With regard to the incidence and history of cardiovascular disease, the results indicate that in patients with high cholesterol, incidence of cardiovascular disease is higher than healthy people, thus indicating a potential genetic link between hypercholstrolemic family and heart disease. The significance of HWE testing in populationbased genetic association studies is immense especially when analyzing the control group. This is because an important assumption underlying these studies is that the control group is a representative sample of the population under investigation. Another assumption in such studies is that individuals of both case and control groups belong to the same single large random-mating population. 

In this study Hardy-Weinberg equilibrium for the alleles studied in rs515135 and rs693 polymorphisms in the *APOB* gene and the unbalance of G and T was established (P>0.05).

## Discussion

Of the theoretical estimated prevalence of 1/500 for heterozygous FH, <1% are diagnosed in most countries. Recently, direct screening in a Northern European general population diagnosed approximately 1/200 with heterozygous FH. All reported studies document the failure to achieve the recommended LDL cholesterol targets in a large proportion of individuals with FH, which may have up to 13-fold increased risk of CHD. Based on prevalences between 1/500 and 1/200, between 14 and 34 million individuals worldwide have FH (22). 

Early detection and treatment probably would reduce premature morbidity and mortality of this disease. Cascade screening of family members of known index cases is the most cost-effective approach for identification of new FH cases (23). Once diagnosed, individuals with FH can be treated with lifestyle measures, lipid-lowering therapies, and possibly novel therapies including PCSK9 monoclonal antibodies, anti-sense oligonucleotides targeting *APOB* and microsomal triglyceride transfer protein inhibitors to change the clinical course of the disease (22).

This is the first study investigating the association of *APOB* polymorphisms with FH. This study provides an analysis of two *APOB* polymorphisms and their correlation with variation in serum lipid levels in the Iranian population. Significant findings were observed for the genetic association between *APOB* (rs515135) and (rs693) polymorphisms with variation in TC genotype levels among the Iranian samples analyzed. Heterozygous samples at the *APOB* rs693 locus were significantly associated with lower TC serum levels .This may suggest an interaction between the two alleles to influence serum TC levels and thus genetically predispose individuals to dyslipidemia.

Univariate analysis of the *APOB* rs693 polymorphism revealed a significant association between carriers of the allele with lower mean serum TC. These abnormalities in lipid profile associated with the *APOB* rs693 polymorphism may be the result of a change in the degree of hydrophobicity and efficacy of *APOB* processing (24-26). Moreover, there was no statistically significant difference in plasma levels of the total cholesterol with respect to the *APOB* rs515135 SNP.

Among our studied population, the rare T allele was observed may be have a “protective” role exhibiting decrease in the risk of high TC levels in individuals homozygous for the rare T allele. Some of the subjects in the present study with positive family history of hypercholesterolemia (n=120) also showed a significant association with the rare T allele where there was a higher frequency of heterozygotes (26.67%). Logistic regression analysis also showed a significantly lower TC levels in individuals with the homozygous TT genotype.

## Conclusion

In this study no significant relationship was found between rs515135 and familiar hypercholesterolemia. However, there was a significant association between the C allele of rs693 and high familial cholesterol levels.
